# Roles of *Drosophila* Hox Genes in the Assembly of Neuromuscular Networks and Behavior

**DOI:** 10.3389/fcell.2021.786993

**Published:** 2022-01-07

**Authors:** Rohit Joshi, Rashmi Sipani, Asif Bakshi

**Affiliations:** ^1^ Laboratory of Drosophila Neural Development, Centre for DNA Fingerprinting and Diagnostics (CDFD), Hyderabad, India; ^2^ Graduate Studies, Manipal Academy of Higher Education, Manipal, India

**Keywords:** Hox, *Drosophila*, motor neuron (MN), behavior, feeding, locomotion, self righting behavior, neuromuscular network

## Abstract

Hox genes have been known for specifying the anterior-posterior axis (AP) in bilaterian body plans. Studies in vertebrates have shown their importance in developing region-specific neural circuitry and diversifying motor neuron pools. In *Drosophila*, they are instrumental for segment-specific neurogenesis and myogenesis early in development. Their robust expression in differentiated neurons implied their role in assembling region-specific neuromuscular networks. In the last decade, studies in *Drosophila* have unequivocally established that Hox genes go beyond their conventional functions of generating cellular diversity along the AP axis of the developing central nervous system. These roles range from establishing and maintaining the neuromuscular networks to controlling their function by regulating the motor neuron morphology and neurophysiology, thereby directly impacting the behavior. Here we summarize the limited knowledge on the role of *Drosophila* Hox genes in the assembly of region-specific neuromuscular networks and their effect on associated behavior.

## Introduction

Feeding, locomotion, and reproduction are some of the most fundamental behaviors exhibited by bilaterians. Regional specialization of the muscles, as well as the central nervous system (CNS) along the anterior-posterior (AP) axis, is a prerequisite for the successful and reproducible execution of these behaviors (Philippidou and Dasen, 2013). Role of Hox genes in assembling region-specific neural circuitry and MN diversification has been examined in vertebrates (Dasen et al., 2005; Dasen et al., 2008; di Sanguinetto et al., 2008; Philippidou and Dasen, 2013) and to some extent in insects (Dixit et al., 2008; Dutta et al., 2010; Baek et al., 2013; Garaulet et al., 2014; Enriquez et al., 2015; Picao-Osorio et al., 2015; Friedrich et al., 2016; Issa et al., 2019; Garaulet et al., 2020). Hox genes are also involved in the specification, survival, and functioning of other neuronal cell types (van den Akker et al., 1999; Pattyn et al., 2003; Gaufo et al., 2004; Holstege et al., 2008; Miguez et al., 2012; Huber et al., 2012; Bussell et al., 2014; Baek et al., 2019); however, such reports (for specific cell types) are limited. The MNs are central players in the functioning of neuromuscular networks. Their role in fine tuning muscle control is important for behavioral execution. The loss of MNs or perturbation of their function owing to a disease affects the behavior and leads to progressive muscle wasting. Therefore, studying their specification and functioning will give insights into the molecular basis of complex behaviors and disease.

In *Drosophila,* Hox genes are known to establish segment-specific patterns of myogenesis and neurogenesis (Michelson, 1994; Technau et al., 2014). However, the molecular basis of how Hox genes play a role in the specification and regional adjustment of the motor neuron (MN) networks is just beginning to be understood. Therefore much remains to be learned about their role in the assembly, maintenance, and functioning of segment-specific neuromuscular networks. In this regard, *Drosophila* as a model organism offers many unique advantages over other models (Hales et al., 2015; Schlegel et al., 2017; Yamaguchi and Yoshida, 2018). These advantages are a short life cycle, a fully sequenced genome, less redundancy than vertebrates, and a wide array of molecular genetic tools. In its short life cycle, *Drosophila* undergoes remarkable morphological and behavioral changes with different modes of feeding and locomotion for different stages. In just 10 days, it progresses from a static, non-feeding embryonic stage to a crawling and feeding larva, followed by an immobile and non-feeding pupal stage, eventually eclosing as a sexually active adult with an entirely different mode of navigation, locomotion, and foraging. It has a wide repertoire of simple, well-established behaviors (Nichols et al., 2012; Neckameyer and Bhatt, 2016), and many of the neuromuscular modules executing these behaviors are simple and well investigated. Compared to the vertebrates, *Drosophila* has a relatively less complex nervous system and musculature, and a fantastic array of molecular tools for reproducibly making subtle genetic manipulations in a cell-specific manner. The effect of these manipulations can be assayed in live and behaving animals (Korona et al., 2017; Martín and Alcorta, 2017), which is a tremendous advantage in correlating a gene to behavior.

In this review, we summarize existing *Drosophila* literature elucidating the role of Hox genes in the assembly and functioning of region-specific muscle-MN connections and their contribution in executing associated behaviors.

## Role of Hox Genes in the Specification of Anterior-Posterior Axis in *Drosophila* Central Nervous System

**FIGURE 1:**
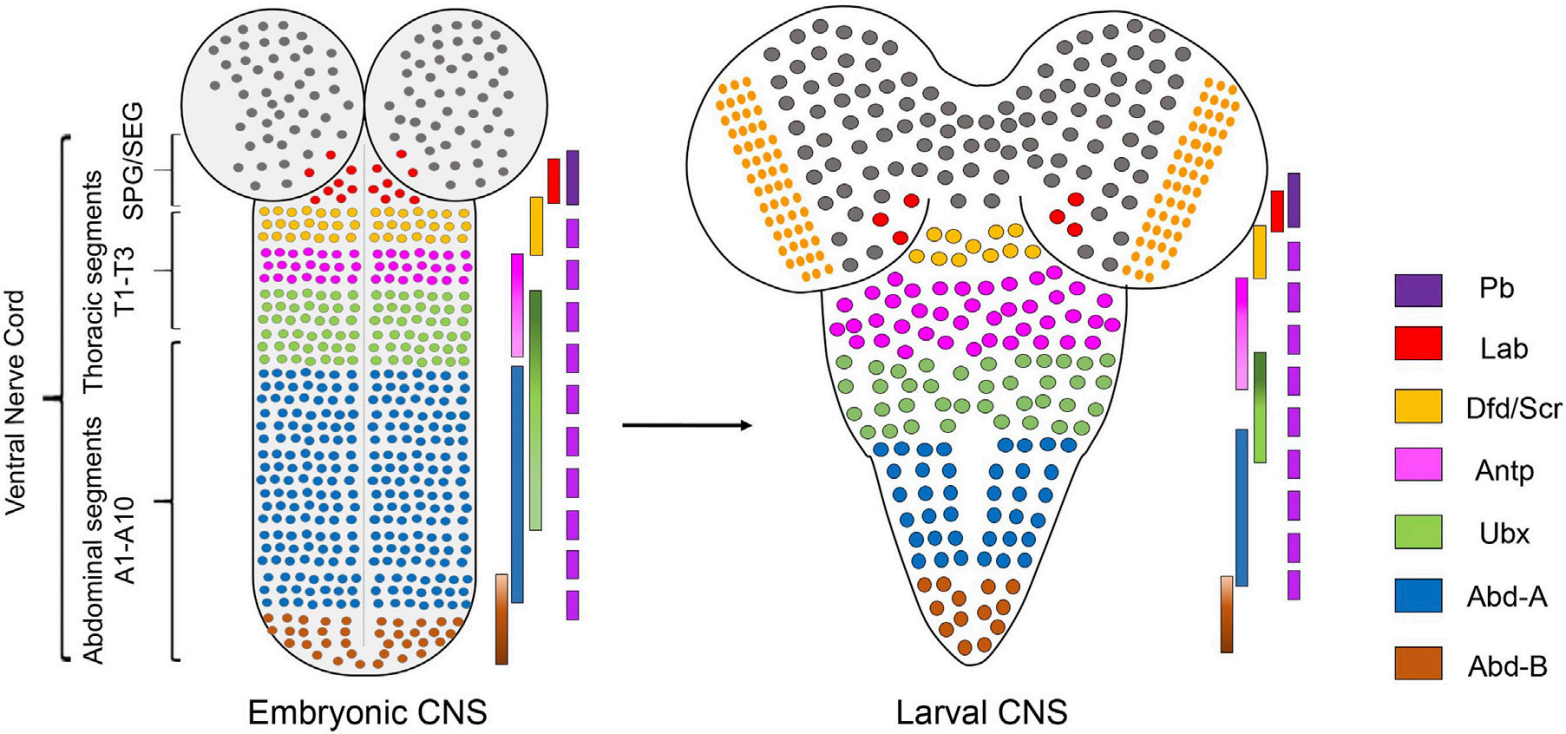
Expression of Hox genes in *Drosophila* CNS. Schematics of embryonic (stage 14) and second instar larval CNS show Hox genes’ expression pattern in different regions along the AP-axis. *Drosophila* CNS has a brain and ventral nerve cord (VNC). VNC is divided into SPG/SEG region, thoracic segments (T1-T3), abdominal (A1-A7) segments, and terminal (A8-A10) segments. The precise extent of Hox gene expression in these regions is shown by overlapping color-coded bars. Pb is expressed in all the segments from SEG to A9.

Hox genes are a family of homeodomain (HD) containing transcription factors (TFs), which play an important role in determining the anterior-posterior (AP) axis of bilaterian organisms (Hart et al., 1985; Regulski et al., 1985; Akam, 1989; Carroll, 1995). They are known to specify the AP axis by differentially regulating their downstream target genes with the help of TALE-HD containing cofactors Pbx/Exdradenticle (Exd) and Meis/Homothorax (Hth) (Mann and Chan, 1996; Mann and Affolter, 1998; Moens and Selleri, 2006; Merabet et al., 2007; Lelli et al., 2011; Saadaoui et al., 2011; Hudry et al., 2012). Hox genes execute these functions by giving the segments where they are expressed a very distinct identity, translating into divergent morphologies/properties along the AP axis of the body (including epidermal structure, CNS, and musculature). In *Drosophila,* there are eight Hox genes (compared to 39 Hox genes in vertebrates) which are organized into two complexes-the Antennapedia Complex (Antp-C) (Kaufman et al., 1990) [comprising the genes *labial (lab)*, *proboscipedia (pb)*, *Deformed (Dfd)*, *Sex combs reduced (Scr)*, *Antennapedia* (*Antp*)]*,* and the Bithorax Complex (BX-C) (Sánchez-Herrero et al., 1985; Tiong et al., 1985; Maeda and Karch, 2006) [consisting of the genes *Ultrabithorax (Ubx)*, *abdominal-A* (*abd-A)* and *Abdominal-B (Abd-B)*]. *Drosophila* CNS consists of the brain and segmented ventral nerve cord (VNC). Hox genes pattern the VNC, which is the *Drosophila* equivalent of the vertebrate hindbrain and spinal cord. The embryonic VNC specified by Hox factors can be broadly divided into five regions, namely: supra-esophageal ganglia (SPG) expressing *pb* and *labial*; sub-esophageal ganglia (SEG) [composed of maxillary (Mx), mandibular (Mn), and labial segments (Lb)] expressing Hox genes *Dfd, Scr* and *Antp*; thoracic ganglia (T1-T3 segments) expressing *Antp* and *Ubx*; abdominal ganglia (A1-A7 segments) expressing *Ubx*, *abd-A,* and *Abd-B*; and the terminal region (A8-A10 segments) expressing *Abd-B* (Hirth et al., 1998; Kuert et al., 2014) (Figure 1). The expression of *pb* has also been reported in other segments (SEG to A9) of VNC (Baek et al., 2013; Enriquez et al., 2015; Hirth et al., 1998).

The neurogenesis in *Drosophila* happens in two phases, embryonic and larval, separated by a period of mitotic quiescence for the neural stem cells (called neuroblast-NB), which are the progenitors and generate all the neurons and glial cells of the CNS (Homem and Knoblich, 2012). In embryonic stages (stages 9–11), NBs delaminate from the neuroectoderm in each segment (Hartenstein and Wodarz, 2013). Five such successive waves of delamination generate 30 NBs per hemisegment of the embryo (Truman and Bate, 1988; Doe, 1992; Hartenstein and Wodarz, 2013). This blueprint of the CNS, when superimposed with the spatial genes [responsible for determining the AP and DV (Dorso-Ventral) (Skeath, 1999) axis and segment polarity genes (Bhat, 1999)], gives the NBs their specific positional identity (Schmid et al., 1999; Truman et al., 2004). This spatial identity of the NBs, in collaboration with the sequentially expressing temporal series TFs (Doe, 2017) expressed during embryogenesis, results in the generation of a segment-specific variety of cell types and cell numbers in the embryo. The transition of these temporal series TFs (Hunchback>Kruppel>Pdm>Castor >Grh) is intricately coupled to the NB cell cycle, which precisely times the expression of these factors and further contributes to specific cell type generation (Isshiki et al., 2001). The embryonic phase generates neurons required for larval CNS and eventually contributes to 10% of the adult neurons, while postembryonic neurogenesis contributes to the remaining 90% of the adult neurons (Homem and Knoblich, 2012). Hox genes contribute to the generation of the cellular variety along the AP axis in both embryonic and post-embryonic phase of neurogenesis by regulating fate specification, quiescence, proliferation, differentiation and apoptosis of NBs and their progeny (Prokop and Technau, 1994; Prokop et al., 1998; Bello et al., 2003; Miguel-Aliaga and Thor, 2004; Berger et al., 2005a; Berger et al., 2005b; Rogulja-Ortmann et al., 2008; Tsuji et al., 2008; Kannan et al., 2010; Karlsson et al., 2010; Suska et al., 2011; Kuert et al., 2012; Baek et al., 2013; Birkholz et al., 2013a; Birkholz et al., 2013b; Estacio-Gómez et al., 2013; Baumgardt et al., 2014; Kuert et al., 2014; Arya et al., 2015; Becker et al., 2016; Khandelwal et al., 2017; Monedero Cobeta et al., 2017; Yaghmaeian Salmani et al., 2018; Bahrampour et al., 2019; Ghosh et al., 2019; Bakshi et al., 2020).

In the CNS, Hox genes are expressed in NBs in early embryonic stages, but their expression from the NBs is largely excluded thereafter. However, Hox genes continue to express in the neurons as they differentiate, project axons/dendrites, and form synaptic connections (Hirth et al., 1998) in embryonic and postembryonic stages. This led to the suggestion that Hox genes may have a role in the assembly and functioning of neuromuscular networks (Hirth et al., 1998; Dixit et al., 2008). More so, since the regionally distinct muscle patterns are known to be established by Hox genes early in development (Michelson, 1994). However, barring their role in regulating neuronal differentiation and apoptosis (Hirth et al., 1998; Miguel-Aliaga and Thor, 2004; Rogulja-Ortmann et al., 2008; Karlsson et al., 2010; Suska et al., 2011; Baek et al., 2013; Estacio-Gómez et al., 2013), the utility of the sustained expression of Hox genes in neurons had not been entirely clear. Therefore, the role of Hox genes in the regional specialization of the MNs, and their contribution to the assembly of functional neuromuscular networks (along the AP axis) remained unaddressed for a long time. Here, we focus on the reported roles of *Drosophila* Hox genes in the establishment of functional neuromuscular networks and behavior (summarized in Table 1). We also attempt to identify some common themes in the context of neuromuscular network assembly and functioning, which are independent of the conventional role of Hox genes in AP axis determination.

**TABLE 1:** Role of Hox genes in establishing neuromuscular networks and behaviour in *Drosophila*.

Function	Hox gene involved	Location of action of Hox	Specific roles of Hox	References
Peristaltic movement in larval locomotion (Figure 2A)	Ubx/AbdA	Muscles and neurons	Establishing region specific neuromuscular networks. Region-specific matching of MN and Muscle (suggested)	Dixit et al. (2008)
Establishment of neuromuscular network for adult legs (Figures 2B,C)	Scr/Antp/Ubx	Thoracic LinA MNs	LinA MN survival	Baek et al. (2013)
			Axonal targeting of LinA MNs and innervation of leg muscles	
			Axonal arborisation on leg muscle	
			Antp level dependent axonal targeting to proximal and distal leg regions	
Adult locomotion	Pb	Thoracic LinB MNs	Regulation of axonal and dendritic morphology with the help of mTFs	Enriquez et al. (2015)
			Targeting of 3 LinB MNs that innervate leg muscles	
			Controlling the walking stance of the adults at high speed	
Larval feeding (Figure 3)	Dfd	MHE muscles and maxillary nerve motor neurons	Regulation of axonal outgrowth of MNs from the SEG that innervate the MHE muscles	Friedrich et al. (2016)
			Formation and maintenance of synapses at the NMJ in the MHE by regulation of molecules controlling synaptic specificity	
Establishment of embryonic muscle innervation pattern (Figure 4)	Ubx	VL2 muscles and VL1 MNs	Regulation of Wnt4 and Sulf-1 in VL2 muscles that signal and repel away approaching growth cones of VL1 MNs	Hessinger et al. (2017)
			Controlling expression of target genes in VL1 MNs to repel VL1 MNs from VL2 muscles	
Female egg-laying	Ubx	Fru^+^ neurons	Proper oviduct innervation by Fru^+^ ILP7 MNs	Garaulet et al. (2014)
			Maintenance of MN synapses on oviduct and radial muscles	
Larval Self-righting behavior (Figures 5A,B)	Ubx	Larval SR node MNs	Regulation of neural Ca^2+^ activity of the SR node MNs	Picao-Osorio et al. (2015)
Adult Self-righting behavior (Figures 5C,D)	Ubx	Adult SR node MNs (these are distinct from larval SR MNs)	Regulation of neural Ca^2+^ activity of the SR node MNs	Issa et al. (2019)
			Maintenance of synaptic structures on the adult leg muscles	

## Role of Hox Genes in Locomotion

One of the first reports implicating the role of Hox genes in assembling the segment-specific neuromuscular networks in *Drosophila* was by Dixit et al. (2008) from Bate and Vijay Raghavan groups in Cambridge and Bangalore. This report established the role of Hox genes in regulating segmental peristaltic movements in larval locomotion (Dixit et al., 2008). This work, in many ways, laid the foundation for exploring the molecular basis of the genetic control that results in equivalent cells of CNS (along the AP axis) to form regionally specialized neuromuscular networks.

**FIGURE 2:**
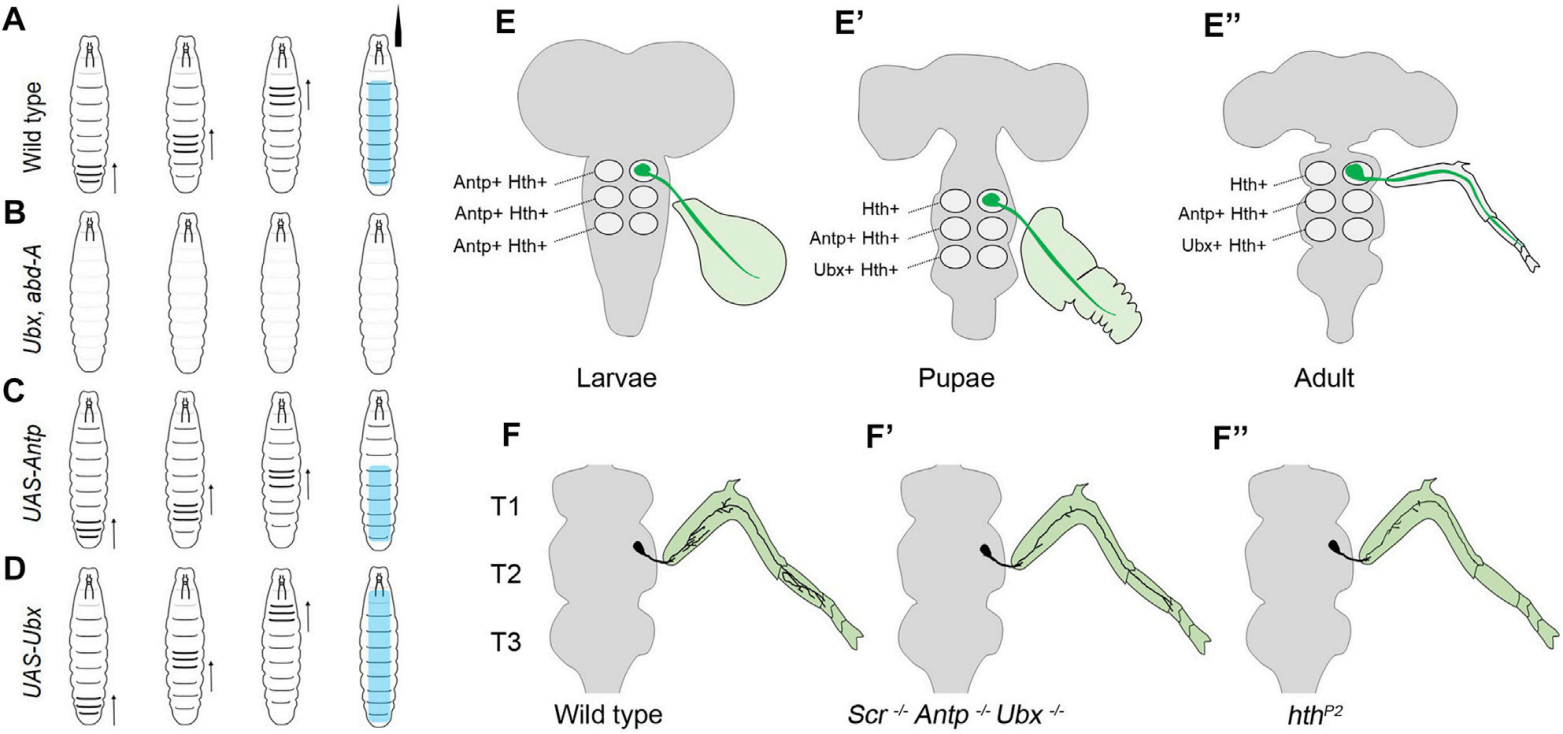
Summary of the role of Hox genes in larval peristalsis and leg innervating MNs. **(A)** Schematic showing abdominal peristaltic movement in wild-type larva. **(B)** Shows that abdominal peristaltic movements are lost in *Ubx, abd-A* double mutant. **(C)** Shows that Antp overexpression transforms anterior segments but transformed segments do not show peristaltic movements. The abdominal peristaltic movements are unaffected. **(D)** Shows that in the case of *Ubx* overexpression*,* thoracic and more anterior segments get transformed and gain abdominal peristaltic movements. The direction of peristalsis is shown with an arrow, and its extent is shown in cyan color. **(E–E”)** Shows that thoracic MNs (in green) innervate primordial leg tissue in larval and pupal stages and adult leg muscles. Also shown is the change in the expression code of Antp, Ubx, and Hth in LinA MNs of different thoracic segments (T1-T3) across different developmental stages. **(F–F”)** Shows the wild-type arborization pattern of thoracic MNs innervating to the adult leg muscles. This axonal arborization is affected in triple Hox triple mutants (*Scr* ^*−*^, *Antp* ^*−*^, *Ubx* ^*−*^) and *hth* ^*P2*^ mutants. This suggests that Hox/Hth is required for the survival, targeting and morphology of MNs innervating to the adult leg.

This study showed that the thoracic and abdominal segments of the larval body have distinct movement patterns during larval peristalsis (Figure 2A). The experiments suggested that the abdominal peristaltic movements critically relied on BX-C, specifically *Ubx* and *abd-A* (Figure 2B). The mutants for *Ubx* or *abd-A* were used to show that either of these genes was necessary for developing the neuromuscular networks coordinating these movements. Interestingly, the ubiquitous expression of either of the two genes was also sufficient for transforming the anterior segments to posterior identity (Figure 2D). This resulted in morphological transformation of the epidermal denticle belts (which had been known from earlier studies) and the anterior segments gaining the peristaltic activity like abdominal ones. However, this did not happen in the case of Antp overexpression (Figure 2C). In the absence of anatomical data, the study speculated that these movements rely on region-specific muscle architecture and their precise innervation by cognate MNs. In agreement with this, the expression of *Ubx* or *AbdA* in muscles was not sufficient for anterior segments to show a peristaltic pattern similar to posterior segments. This observation supported the idea that in the case of Hox-dependent segmental transformation, the MNs and the entire neural circuitry are reorganized to match the transformed pattern of muscle. This study also suggested that a one-to-one match in the identity of the muscle with that of the underlying neural circuitry is required for the proper execution of abdominal peristaltic movements.

Subsequently, two studies comprehensively addressed the developmental role of Hox genes in survival, targeting, and morphology of thoracic MNs, which innervate the leg muscles responsible for adult locomotion (Baek et al., 2013; Enriquez et al., 2015). These studies were built upon previous work which had shown that 50 MNs innervating the *Drosophila* adult leg muscles are generated by 11 NBs located in each thoracic hemisegment (Baek and Mann, 2009; Brierley et al., 2012). Two-third of these 50 MNs are generated by two NB lineages, LinA (or Lin 15), which generate 28 MNs, and LinB (or Lin 24), which generate seven MNs (Truman et al., 2004; Baek and Mann, 2009). These studies had characterized stereotypic axonal and dendritic morphologies of all the 50 MNs (generated by LinA and LinB) at the single-cell level, down to their synaptic innervations of the 14 leg muscles on the four segments of the adult leg (Baek and Mann, 2009; Brierley et al., 2012).

The first study by Baek et al. (2013), from Mann’s group in New York focussed on LinA MNs and showed that Hox genes (*Scr*, *Antp*, and *Ubx*) and their TALE-HD containing cofactor Hth are required for the survival of the MNs in all the three thoracic segments. They found that all newly born thoracic MNs express Antp and Hth in all the three thoracic segments in larval stages (Figures 2E–E'). As the development progressed, this expression code transformed from being Antp^+^/Hth^+^ in all the segments to Hth^+^ in T1, Antp^+^/Hth^+^ in T2, and Ubx^+^/Hth^+^ in the T3 segment in the late pupal stage and adults (Figures 2E–E'). This change in TF code is suggested to specialize the MNs innervating the adult legs to execute their segment-specific functions (Szebenyi, 1969; Kaplan and Trout, 1974; Dawkins and Dawkins, 1976; Trimarchi and Schneiderman, 1993; Dickinson et al., 2000). Exd was found to express in all the segments in all the stages. Subsequent clonal analysis (Wu and Luo, 2006) done with *Hox triple* (*Scr* ^*−*^, *Antp* ^*−*^, *Ubx* ^*−*^) and *hth* mutants indicated that expression of both *Hox and hth* genes is required autonomously within the thoracic lineages for survival, targeting, and morphology of the adult MNs (Figures 2F–F') (Baek et al., 2013). Since Antp was the only Hox factor to be expressed initially in NBs, *Antp* single mutant was tested. Curiously, this mutant recapitulated most of the phenotype exhibited by *Hox triple* mutant, except the death of MNs in the T3 segment, which was attributed to *Ubx* and *Antp* redundancy in this segment. Subsequently, the death of the MNs was blocked by the expression of p35, a viral protein commonly used to block apoptosis (LaCount et al., 2000). In this case, the surviving MNs in *Hox triple* (*Scr* ^*−*^, *Antp* ^*−*^, *Ubx* ^*−*^) or *hth* mutant backgrounds targeted roughly to the same region along the proximal-distal (PD) axis of the adult leg segments, with terminal arborization defects. This suggested that *Hox* (and *hth*) genes are not needed by LinA progeny to assume the thorax-specific lineage identity or the MN fate. However, they are required for the appropriate specification of the finer morphological features of these MNs necessary for the functional muscle innervation (Figures 2F–F'). This was similar to what is known for the role of Hox genes in vertebrates MN specification, wherein MN identity is established independent of Hox genes (Jessell, 2000; Dasen et al., 2005).

The study also provided a novel alternative mechanism to diversify cell fate within a given lineage by modulating the expression level of Hox factor Antp. Usually, NB progeny rely on temporal series TFs for fate diversification (Doe, 2017). Baek et al. (2013) observed that within the same lineage, Antp is expressed at high levels in late-born MNs and low levels in early-born MNs. This variation in the Antp gene expression levels in MNs was found to have an instructional role in their axonal targeting. It was observed that high Antp expressing late-born MNs targeted the distal region, and low Antp expressing early-born MNs targeted the proximal regions of the adult leg. Expectedly, this pattern could be reversed by overexpression or the knockdown of *Antp*. Though, in the null allele of *Antp,* both distal and proximal targeting of MN was affected with no specific bias, indicating that low level gave a distinct phenotype from the absence of *Antp*. This variation in the expression levels of *Antp* had cell-autonomous consequences in MN innervation and did not show any defect in the leg muscles of the adult fly. This was in contrast to an earlier work by Dutta et al. from VijayRaghavan and Rodriguez groups at Bangalore, where Hox dysregulation in MNs resulted in muscle development defects (Dutta et al., 2010). The experiment in this study shows that knockdown of *Ubx* in adult MNs resulted in modest reduction and developmental deformity in adult leg muscles (Dutta et al., 2010). On the other hand, *Ubx* overexpression in the MNs innervating thoracic dorsolateral muscle (DLM) of adults caused a dramatic reduction in the number of DLM fibers (Dutta et al., 2010). These observations implied an active communication between the adult thoracic MNs and their muscle targets. Dutta et al. (2010) also suggested that Hox expression needs to be tightly controlled within a narrow range for the assembly of functional neuromuscular network. It is to be noted that this study relied on a chronic knockdown/overexpression of *Ubx* in the MNs, while Baek et al. (2013) used temporally controlled overexpression or knockdown.

The requirement of Hox genes in determining the morphology of thoracic MNs was followed up by Enriquez et al. from Mann’s group in New York (Enriquez et al., 2015). This work identified the role of Hox gene *proboscipedia (pb)* in determining the morphological characteristics of three thoracic MNs (Enriquez et al., 2015). The *proboscipedia (pb)* expresses from the supraesophageal region to the A9 segment in embryonic and larval CNS (Hirth et al., 1998; Baek et al., 2013), but its role in neurogenesis had not been tested. Previous studies had suggested that the morphology of MNs arising out of thoracic LinA and LinB lineages were under precise genetic control, which had a bearing on their function (Baek and Mann, 2009; Brierley et al., 2012). However, the genetic determinants regulating the individual neuronal morphology for these (or any neuron) were not known. LinB (or Lin 24), which produces only seven MNs in each hemisegment (of 3 thoracic segments) were an attractive system to address this problem owing to few but well-characterized MNs in this lineage (Baek and Mann, 2009; Brierley et al., 2012). Enriquez et al. screened 230 antibodies against different TFs for their expression in larval LinB lineage [marked by GFP using MARCM (Wu and Luo, 2006)]. They identified six TFs whose combinatorial expression was sufficient to uniquely classify the seven MNs of LinB lineage. These factors were Empty spiracle (Ems), the Zinc finger homeodomain factors 1 and 2 (Zfh1 and Zfh2), the Hox TF Proboscipedia (Pb), the Pax6 ortholog Twin of Eyeless (Toy), and Prospero (Pros). Further, they observed that the TFs combinations observed in each of the seven MNs were not observed in any other neuron of the CNS. After that, they used lineage tracing experiments to correlate larval LinB MNs (with unique TF code) to their adult counterparts, each of which corresponds to distinct morphology and muscle innervation. This supported the idea that the characteristic expression of these six TFs probably results in distinct axonal and dendritic morphologies of these MNs. Consequently, these factors were called morphology TFs (mTFs).

Next, a clever combination of MARCM (Wu and Luo, 2006) with the Flybow technique (Hadjieconomou et al., 2011) was used to mark adult MNs where they removed or overexpressed *pb* to analyze its effect in MNs of LinB. The Hox gene *pb* was shown to be essential for the morphological identity of 3 out 7 MNs of LinB. Interestingly, loss or overexpression of *pb* did not affect the expression of the other five mTFs, which was in contrast to what is known for the temporal series TFs (Doe, 2017), which play a crucial role in generating neuronal diversity. When *pb* mutant LinB NB was analyzed, it was observed that the number of MNs generated in the lineage was unaffected. These MNs did not lose or change their identity; they remained glutaminergic, and their axons targeted the leg muscles. However, there was a reduction in the area covered by dendrites of MNs, and specific axon targeting defects were observed on adult leg muscles. Conversely, misexpression of *pb* in LinA MNs resulted in the relocalization of their dendrites to an area on the neuropil, where typically LinB dendrites were located. However, LinA retained many of its features and did not gain all the characteristics of LinB. Since *pb* mutant MNs show defective leg muscle innervation, the adults with *pb* mutant MNs were tested for walking behavior. Most walking parameters were normal, except that at high speed, the flies with *pb* mutant LinB MNs showed more wobble in walking than the control adults. This indicated a role of Pb expressing LinB MNs in stable walking at high speed. In order to establish that mTF code (of Pb with other factors) was instructive for the MN morphology, the TF code of specific LinB MNs was changed to other MNs in the LinB lineage by simultaneous knockdown and overexpression of the mTFs. It was observed that altering the mTF code resulted in the predictable transformation of the morphology, which supported the idea that different combinations of mTFs determined the MN’s morphological identity and led to the suggestion that role of *pb* in morphology had a critical bearing on the fly walking behavior. These results also established a genetic basis for the morphology of the MNs. They also suggested that MNs rely on a unique combination of different mTFs, which collectively give them their distinct signature morphologies. An idea proposed in the study is that temporal TFs most likely direct a stepwise change in the mTFs code for successive MNs (generated in LinB) and thus progressively change their morphology. To test this idea, it will be an important (though tedious) task to delineate the role of individual mTFs in determining the final MN morphology in the context of LinB. The results also raise the question of whether Pb plays a similar role in determining the morphology of other thoracic MNs (working with a different set of mTFs). Alternatively, considering Pb expression in other segments; it is a possibility that Pb may contribute to determining the morphologies of MNs found in other segments of VNC as well.

Moreover, since different levels of Antp have already been shown to play a role in regulating the morphology of MNs (Baek et al., 2013), one wonders if there is any interaction between *pb* (or other mTFs) and resident Hox gene in determining the final MN morphology. It is to be noted here that Antp expressing LinA MNs did not express Pb (Baek et al., 2013). Experimental testing of this idea will also determine whether the identity of the NB has any consequence on the choice of the mTFs employed. However, the existence of a lineage-specific combination of the mTFs has already been ruled out by Enriquez et al. (2015).

## Hox Code for Neuromuscular Assembly in Embryogenesis and Larval Feeding Circuit

Feeding is a fundamental behavior necessary for the survival of an animal. In *Drosophila,* the feeding behavior has been investigated in larval and adult stages (Pool and Scott, 2014; Miroschnikow et al., 2020). The neurons responsible for feeding behavior and taste perception reside in the maxillary (Mx) and mandibular (Mn) neuromeres of larval SEG, which express Hox gene *Dfd* (McGinnis and Krumlauf, 1992; Hirth et al., 1998; Kuert et al., 2014). The *Dfd* loss of function mutants die during embryogenesis due to their inability to hatch. The hypomorphic alleles that survive until adulthood starve to death, owing to their inability to extend proboscis and ingest food (Merrill et al., 1987; Regulski et al., 1987; Restifo and Merrill, 1994).

**FIGURE 3:**
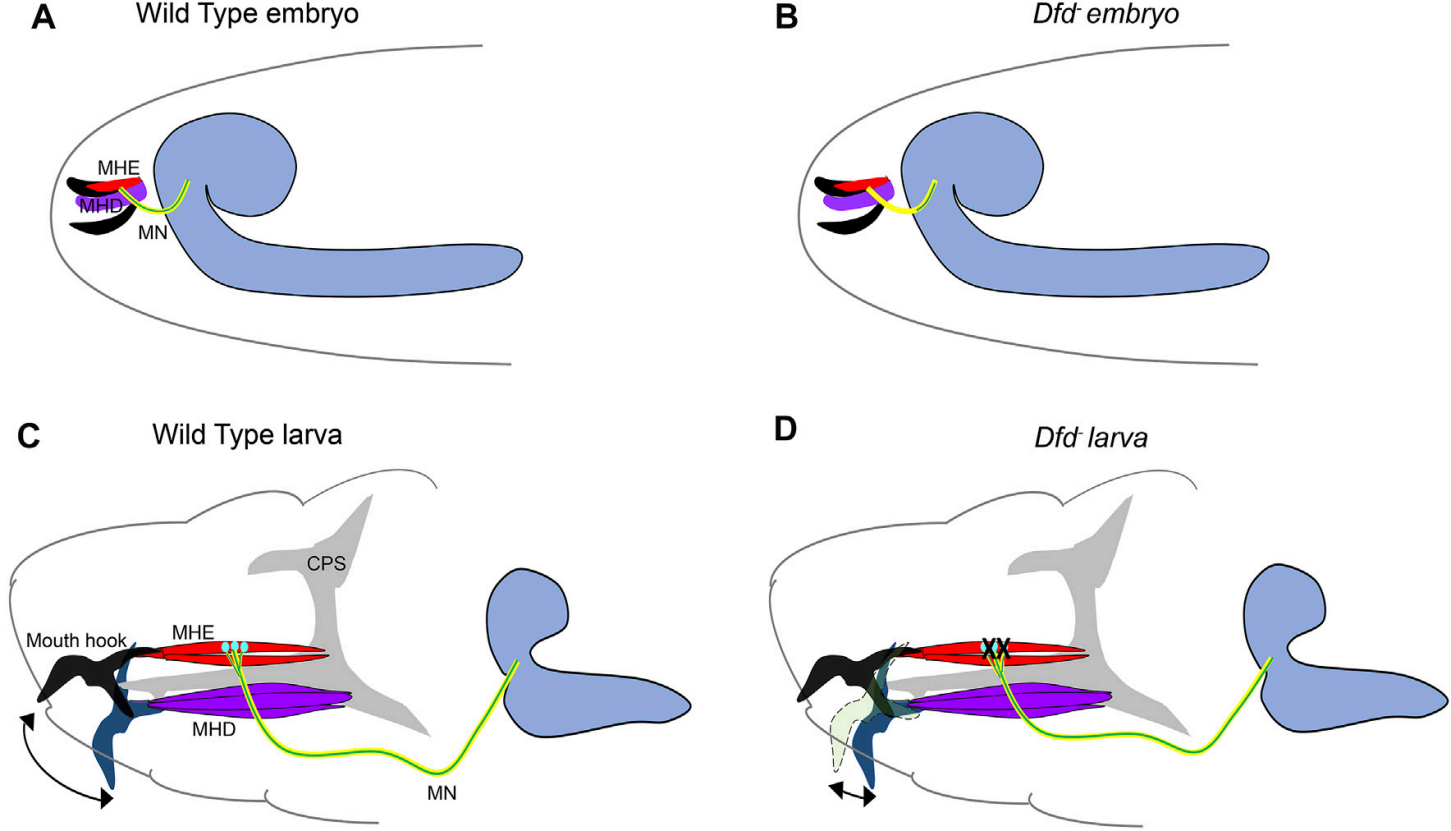
Role of *Dfd* in embryonic axonogenesis and larval feeding. Schematics show the major components responsible for embryonic mouth hook movement and larval feeding circuitry. The maxillary nerve (shown in yellow, originates from the SEG region of embryonic/larval CNS and innervates both MHD (shown in purple) and MHE (shown in red) muscles. The *Dfd* positive motorneurons (shown in green) from SEG synapse with the MHE muscles only and are crucial for the mouth hook elevation during embryonic hatching and larval feeding. The grey shaded region represents the Cephalopharyngeal Skeleton (CPS), extension and retraction of which is integral to the feeding process. The CNS is shown in blue on the right. The mouth hooks are shown in black in the embryos. In larvae, black mouth hook shows the extent of its elevation in wild-type, blue mouth hook shows the extent of its depression in wild type, and light green mouth hooks show the extent of its elevation in a *Dfd* ^*3*^ mutant. **(A,B)** Shows the schematic of wild type and *Dfd* mutant embryos, latter show a severe restriction in axon outgrowth for the Dfd positive motor neurons resulting in failure of these neurons to project to the MHE. These mutants show hatching defects. **(C,D)** Shows the schematic of the wild type and *Dfd* mutant larvae. In the absence of *Dfd*, the mouth hook elevation is drastically reduced (double-sided arrow indicates the extent of mouth hook elevation and depression in wild type and *Dfd* mutant larvae). *Dfd* is required in both MHE and MNs to regulate assembly and maintenance of the feeding motor unit to execute optimum mouth hook movement.

Building on these observations, a study by Friedrich et al. from Lohmann’s group at Heidelberg investigated the role of Dfd in larval feeding behavior (Friedrich et al., 2016) and exploited the fact that both hatching and feeding rely on the same motor circuit responsible for the up and down movement of the larval mouth hooks (Pereanu et al., 2007; Schoofs et al., 2010; Hückesfeld et al., 2015) (Figure 3). These movements are executed by mouth hook elevator (MHE) and depressor (MHD) muscles in the larval head, which receive synaptic input from neurons in Mx, Mn, and Lb neuromeres and contribute to the larval feeding circuit (Pereanu et al., 2007; Schoofs et al., 2010; Hückesfeld et al., 2015). The authors show that the MNs from SEG expressed Dfd and innervate the elevator but not the depressor muscles (Figure 3A). Congruent to these observations, embryos mutant for *Dfd* were found to exhibit axonogenesis defects (Figure 3B) and consequent failure to hatch into larvae.

Blocking the synaptic transmission in Dfd expressing MNs using tetanus toxin also compromised embryonic hatching. Next, using a temperature-sensitive allele (*Dfd* ^*3*^) it was shown that Dfd is chronically required in the assembly, maintenance, and functionality of the feeding circuit. It was observed that *Dfd* ^*3*^ embryos exhibited mouth hook movement and hatching defects when raised to non-permissive temperature in late embryogenesis (which is much after the formation of synapses). In corroboration to this, *Dfd* ^*3*^ larvae, when shifted to non-permissive temperature as late as in the third instar stage, showed head-mouth hook movement defects, further establishing the chronic requirement for the gene (Figure 3D). Interestingly in both these cases, the innervation of the elevator muscle was found to be normal. Similar knockdown (KD) of Dfd in neurons by RNA interference or *Dfd* ^*3*^ allele exhibited a significant change in synaptic morphology coupled with the reduction in the expression of a synaptic gene, Ankyrin-2 extra-large (Ank2-XL). However, unlike in the case of Ubx KD in MNs (Dutta et al., 2010) reported earlier by Dutta et al., muscles in the larval feeding circuit were normal in the case of Dfd KD in MNs. Since Dfd was found to be expressed both in the elevator muscles and the MNs from SEG, it was proposed that Hox expression in both these cells types provides them with a molecular code to identify each other during synaptic assembly. In agreement with this idea, a synaptic target recognition molecule “Connectin” (*Con*) (Nose et al., 1994) was found to be amongst the direct transcriptional targets of Dfd in CNS. Interestingly, this homophilic cell adhesion molecule Con was expressed in MNs and muscle devoid of Dfd protein, and its expression was regained in the MNs mutant for *Dfd*. This suggested that Dfd functioned to bring together MNs and cognate muscles by actively repressing *Con* in the cells of the feeding circuit. However, the identity of cell adhesion molecule(s) positively required by these cells for assembly of the neuromuscular feeding unit is yet to be determined.

The work established the role of Dfd as a critical coordinator for the formation, maintenance, and functioning of the neuromuscular network in the larval feeding circuit. The results also showed that synaptic stability and plasticity are determined by the half-life of synaptic proteins as well as the transcriptional program, which sustains the supply of synaptic components that maintains the neuromuscular junction. Lastly, it was proposed that Hox genes provide the molecular code for matching the MNs and muscles during developmental synaptic assembly through their transcriptional targets. However, even though Dfd was shown to have a role in the functioning of the feeding circuit, it remains to be investigated whether Dfd played a role in regulating the neural activity of the motor neurons to regulate the feeding behavior.

**FIGURE 4:**
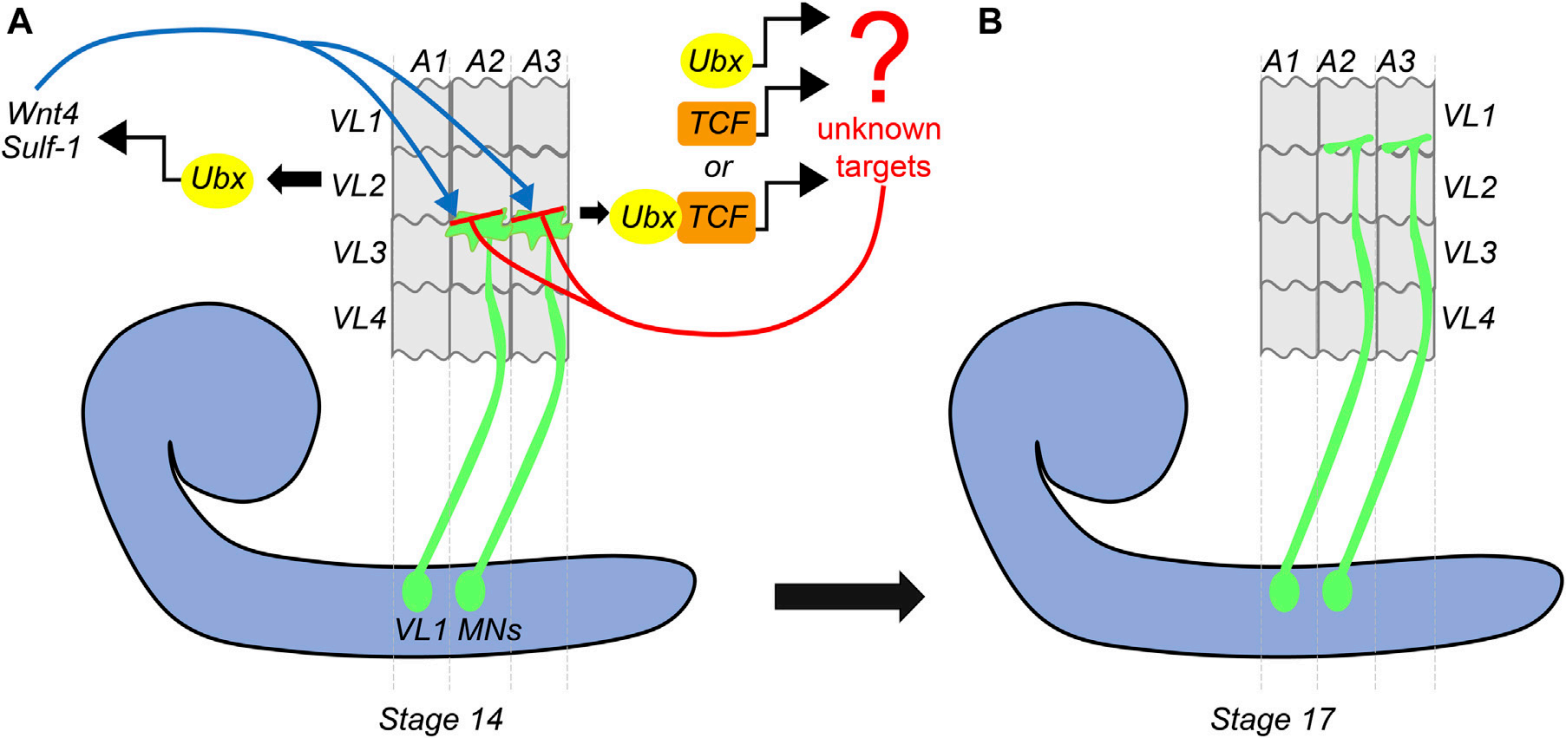
Dual role of Ubx in embryonic muscle innervation. Schematic of stage-14 and 17 embryos show the mechanism of innervation of ventrolateral muscles (VL1-4) of the embryonic body wall (shown in grey) by VL1-MNs (shown in green) coming from the embryonic CNS (shown in blue). **(A)** At stage-14, the approaching growth cone of the VL1 MNs is repelled by Ubx expressing VL2 muscles. Ubx mediates activation of Wnt4 and Sulf-1 in the VL2 muscles, which then interact with the Wnt receptors on the growth cone of the MNs. This leads to the activation of Wnt signaling (armadillo/TCF) in the VL1 MNs. Ubx and TCF in these MNs act together or in parallel to regulate the expression of unknown target genes, resulting in the repulsion of the VL1-MNs by VL2 muscles, thereby pushing them to their final target (VL1 muscles) by stage 17. **(B)** This suggests that Ubx expression in both VL2 muscles and VL1-MNs is required for establishing precise neuromuscular connections in the embryo.

Continuing on the theme of Hox gene providing a molecular basis for matching the MNs and muscles, a subsequent study by Hessinger et al. (2017) from Rogulja-Ortmann and Technau groups at Mainz established a similar role for Hox gene *Ubx* in the assembly of the embryonic neuromuscular junction. This study unraveled the mechanism of how *Ubx* plays a role in determining the target specificity of the MN and its cognate muscle during embryogenesis. In the abdominal segments (A2-A7 segments) of embryonic CNS, ventrally projecting RP MNs innervate ventrolateral (VL) muscles on the embryonic body wall. The RP MNs 1, 3, 4, and 5 are some of the MNs known to innervate four VL muscles (VL1-4) in the abdominal segments (Bossing et al., 1996; Landgraf et al., 1997). Hessinger et al. focussed on the innervation of RP5 and V MNs (referred to as VL1-MNs) onto the VL1 muscles of abdominal segments (Figure 4). Through meticulous genetics, the study established that precise innervation of VL1 muscle by its cognate MNs (VL1-MNs) relies on Ubx mediated activation of Wnt4 signaling in VL2 muscle (Figure 4A). The authors found that AbdA had no role in this innervation, which entirely relied on Ubx dependent expression of the *Wnt4* and *sulfatase 1* gene (*sulf1*-known to be necessary for axon guidance) in the VL2 muscle. Wnt4 and Sulf1 expression in VL2 muscle played an instrumental role in repelling the axons of the MNs facilitating them to innervate their correct target, which was the VL1 muscle (Figure 4B). *Wnt4* is a member of the Wnt family of signaling molecules while Sulf1 is a sulfatase implicated in regulating Wnt and BMP gradient in neuromuscular junction (Nose et al., 1994; Inaki et al., 2007). The secretion of Wnt4 and Sulf1 by VL2 was paralleled with the activation of canonical Wnt4 signaling in VL1-MNs. This facilitated the repulsion of MNs away from VL2 muscles, thereby establishing a precise neuromuscular connection (between VL1 muscle and VL1-MNs). Congruent to this, the knockdown of the canonical Wnt4 signaling pathway in the VL1-MNs resulted in their targeting defects. On the expected lines in *Ubx* mutants, *Wnt4* and *sufl1* genes were downregulated in VL2 muscles. Consequently, VL1-MNs could not go past VL2 muscles, and the innervation of VL1 muscles by these MNs was lost. Finally, as was observed in the case of larval peristalsis (Dixit et al., 2008) and feeding circuitry (Friedrich et al., 2016), it was the simultaneous expression of Ubx in both MNs and the muscles which rescued the *Ubx* mutant phenotype. Collectively, these studies highlight the importance of Hox genes in establishing a complementary molecular code between MN and muscles for the functional assembly of the neuromuscular networks.

## Role of BX-C miRNA Mediated Hox Regulation in Behavior

BX-C has a bidirectionally transcribed microRNA (miRNA) locus with two overlapping miRNA’s on the opposite strand (*iab4/8*). This miRNA locus lies between *abd-A* and *Abd-B* and has been shown to target neighboring homeotic genes and results in homeotic transformation on overexpression (Ronshaugen et al., 2005; Bender, 2008; Stark et al., 2008; Tyler et al., 2008). Phenotypically, the homozygous deletion for the miRNA shows sterility and no other significant phenotype (Bender, 2008; Lemons et al., 2012). Recent studies have focussed on the role of this locus in CNS development, sterility, and adult behavior.

## Role of Ubx in Egg-Laying Behavior

One of these studies by Garaulet et al. (2014) from Lai’s group in New York investigated the role of BX-C miRNA in CNS patterning and female sterility. Garaulet et al. demonstrated that in contrast to the embryonic epidermis where AbdA and AbdB repress anterior Hox gene *Ubx*, in larval CNS, it is the BX-C miRNA, that represses the BX-C genes outside their normal domain of expression. The deletion of this locus results in deregulation of Hox genes *Ubx* and *abd-A* and their cofactor *exd* and *hth* in posterior VNC of larval CNS. This was in agreement with what had been reported for this miRNA previously in the embryonic CNS as well (Bender, 2008; Thomsen et al., 2010; Gummalla et al., 2012). The subsequent genetic analysis shows that the sterility phenotype reported in miRNA-deleted females was substantially rescued by heterozygosity for BX-C genes (*Ubx*, *abdA*) and their cofactor *hth*. This effect was recapitulated by the targeted knockdown of *Ubx* in neurons, thereby establishing that deregulation of *Hox* and *hth* genes in neurons is critical for the sterility phenotype. Phenotypically, the ovary morphology in the mutant female flies was normal, and flies were capable of mating. Since the defect seemed to be in the egg-laying, therefore the focus shifted to the oviduct. The oviduct has two kinds of inputs, *Insulin-like peptide 7* (ILP7) expressing excitatory glutaminergic MNs and inhibitory octopaminergic neurons terminating on radial muscles and epithelial linings (Rodríguez-Valentín et al., 2006; Yang et al., 2008; Castellanos et al., 2013). Significantly, the BX-C miRNA deletion did not alter the number or the transmitter identity for the ILP7+ MNs or the octopaminergic neurons. However, there was a reduction in the innervation of ILP7+ MNs on the oviduct and synaptic bouton count of the MNs on the radial muscles. These defects were substantially rescued by heterozygosity of *Ubx* and *abd-A,* but not by *hth*. However, the overexpression of *Ubx* or *hth* specifically in ILP7+ MNs did not recapitulate the sterility. This suggested that the broad de-repression of these genes in CNS was the cause of adult sterility. A search for functional neuronal domain responsible for the sterility was narrowed down to the Fruitless (Fru) expressing neurons (Stockinger et al., 2005) [which include ILP7+ MNs of oviduct as well (Castellanos et al., 2013)]. The Ubx and Hth overexpression in Fru+ neurons resulted in significant female sterility (90% in Ubx and 22% in Hth), suggesting that these neurons contribute to the female egg-laying program. However, other neuronal lineages from Fru expressing domain relevant for fertility and egg-laying behavior were not identified. Quite surprisingly, a subsequent study by the same group with a new deletion allele for BX-C miRNA showed that female flies were normal in their egg-laying behavior and had a functional neuromuscular control at the genital tract (Garaulet et al., 2020). Instead, this study suggested that the miRNA-deleted female had a behavioral shift from a virgin state to a post-mated state. This shift was attributed to the misregulation of *hth* in CNS. However, whether the misregulation for Hox genes (*Ubx* and *abd-A*) play a role in the behavioral shift was not reported. Also, this study did not investigate the innervation of ILP7+ MNs in females homozygous for new miRNA deletion. This suggests that either Hox genes of BX-C have no role in this behavioral shift for the female flies or the same is yet to be investigated.

## Role of *Ubx* in Self Righting Behavior

**FIGURE 5:**
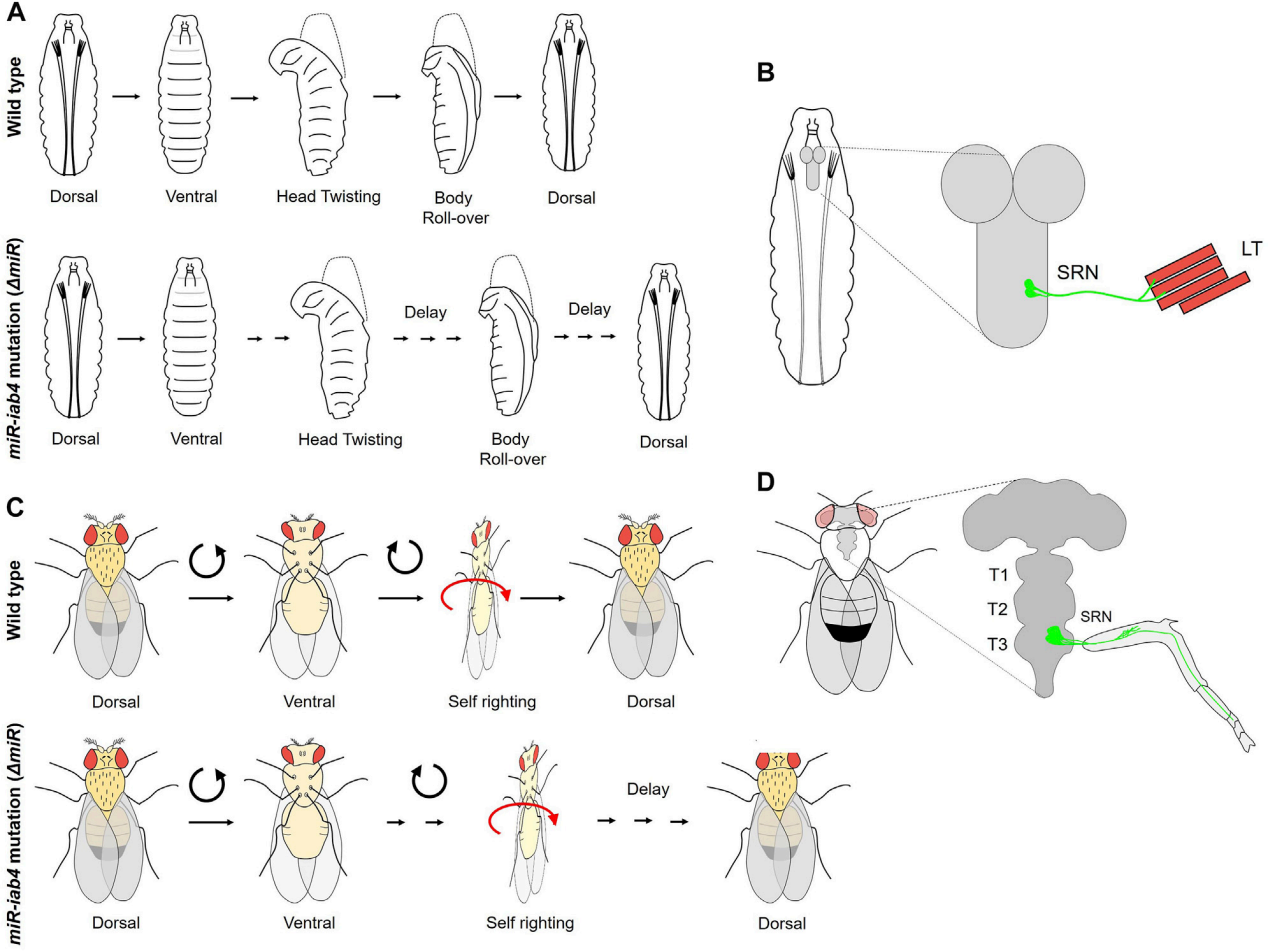
Role of BX-C miRNA in self-righting behavior. **(A)** The larval SR behavioral response in wild-type and *miR-iab4* mutants is shown. Head twisting and body roll-over get significantly delayed in miR-iab4 mutants compared to wild-type larvae. **(B)** Shows that SR node (SRN) neurons originating from abdominal segments innervate to the lateral transverse (LT1/2) muscles of the larval body wall. **(C)** Shows adult self-righting behavioral response in wild type and *miR-iab4* mutants, latter shows delayed SR behavior in adults as well. **(D)** Shows the innervation of the adult leg muscles by SRN neurons originating from T3 segments.

Continuing on the theme of BX-C miRNA mediated repression of homeotic genes, two elegant studies from the Alonso Lab at Sussex in the UK have uncovered a role of the homeotic gene *Ubx* and the BX-C miRNA in self-righting (SR) motor behavior in *Drosophila* larvae and adults (Picao-Osorio et al., 2015; Issa et al., 2019). These studies show for the first time a post-developmental role of Hox gene and the importance of maintaining a very fine control over Hox expression in CNS to regulate neural physiology and behavior. SR is an innate reflex that corrects the body orientation when it is out of its normal upright position (Figures 5A,C). This response is evolutionarily conserved amongst all the bilaterians (Penn and Jane Brockmann, 1995; Faisal and Matheson, 2001; Jusufi et al., 2011).

The first study by Picao-Osorio et al. (2015) established a role of BX-C miRNA *iab4* in the regulation of *Ubx* in a defined group of MNs required to execute the SR behavior in larvae. Initially, the larvae for the deletion of BX-C miRNA were tested for different behavior assays of abdominal peristaltic waves, turning, and SR. All the behaviors were normal except for the SR behavior where miRNA-deleted larvae took a long time to turn themselves over after being put on their dorsal side (Figure 5A). Since *Ubx* was a known target of BX-C miRNA in VNC (Bender, 2008; Tyler et al., 2008; Thomsen et al., 2010; Garaulet et al., 2014), it was tested by targeted overexpression in its native transcriptional domain, and its role was confirmed in SR defects. Next, the cellular basis of aberrant SR behavior was narrowed down to *Ubx* regulation by *iab4* to two metameric MNs in larval VNC (SR node neurons or SRN). The SRN innervated the lateral transverse (LT) muscles of the larval body wall, the LT1/2 (Figure 5B) (Picao-Osorio et al., 2015). Interestingly, the authors did not find any developmental consequence of Ubx dysregulation in larval CNS, and conditional expression of Ubx in the SRN in larval stages could recapitulate the SR behavior defects. This suggested that tampering with the levels of Ubx in these neurons specifically affected physiology and behavior. Similarly, a specific MN GAL4 line, which innervated LT muscles, was used for misexpressing *Ubx* and was shown to delay the SR in larvae. These observations were further corroborated by the differences in calcium activity traces of the SR MNs [measured using *in vivo* calcium sensor GCaMP (Chen et al., 2013)] in miRNA-deleted larvae compared to the wild-type controls. Artificial thermogenic activation (Hamada et al., 2008) or silencing (Kitamoto, 2001) of SR MNs also resulted in SR behavior defects, which was also reflected by the difference in calcium activity traces (Chen et al., 2013) in the test and the wild-type controls. However, it was not clear from this study whether similar SR movements in morphologically distinct organisms like larvae or adults relied on common or different genetic modules. To address this, the same group investigated and found a role of miRNA-mediated *Ubx* regulation in adult SR behavior (Figure 5C) (Issa et al., 2019). In this case, as well, overexpressing *Ubx* in its native domain could recapitulate these defects. Subsequently, *Ubx* was upregulated in two different subsets of adult leg MNs. However, the SR defect was reported in only in one case, further restricting the MNs responsible for SR defects in adults. These MNs were different from those required for executing SR behavior in larvae (Picao-Osorio et al., 2015). The downregulation of *Ubx* in adult-specific SR MNs was sufficient to rescue the behavioral defects reported in miRNA deletion. This knockdown also increased the number of synaptic varicosities on the femur muscles of the adult leg and rescued the neural activity in MNs back to the wild-type levels. These results supported a previously suggested idea that Hox genes have a role in assembling and maintaining the synaptic structures (Friedrich et al., 2016). The *Drosophila* larva and adult are divergent in lifestyle, behavioral properties, muscle structure, and neural constitution. Therefore, this study suggests that similar movements performed by organisms with distinct biomechanical, morphological, and neural structures could rely on the same miRNA/Hox genetic module, which can be redeployed in different developmental stages for equivalent behavior.

Importantly, these studies show that the miRNA-dependent post-transcriptional regulation of Hox gene *Ubx* can control the neural activity of MN to regulate the behavior of an animal. This function of Hox genes in neural physiology is independent of their role in development. The authors also suggest that other behavioral modules (like postural adjustment and locomotion) could also be controlled by miRNA. Furthermore, it is also possible that other adult movement-associated behaviors (like flight, walking, and jumping) (Szebenyi, 1969; Kaplan and Trout, 1974; Dawkins and Dawkins, 1976; Trimarchi and Schneiderman, 1993; Dickinson et al., 2000) may also be regulated by miRNA-mediated regulation of Ubx or Antp. For instance, Baek et al. show that Antp and Hth are the primary factors expressed in all thoracic leg MNs in larval stages. However, in the late pupal and adult stage, the T1 MNs express Hth, T2 express Antp and Hth, and T3 express Ubx and Hth (Baek et al., 2013). Therefore, one possibility worth considering is whether BX-C miRNA-mediated regulation of Antp and Hth also contributes to adult SR or other movement-associated behaviors. This is plausible considering that Hth has already been shown to be a target of BX-C miRNA in CNS (Bender, 2008; Tyler et al., 2008; Thomsen et al., 2010; Garaulet et al., 2014; Garaulet et al., 2020). Since 40% of the miRNA in the *Drosophila* genome were shown to affect the larval SR behavior (Picao-Osorio et al., 2017), therefore it may also be worthwhile to check whether any of these miRNA’s contribute to the regulation of SR or other movement associated behaviors through the regulation of Hox (*Ubx* or *Antp*) or Hth.

Lastly, a tempting question is whether the miRNA/Hox genetic module could also function in MNs of other behavioral circuits like feeding, mating, courtship, grooming, and virgin/mated behavioral shift. Moreover, if such control exists, it needs to be investigated whether it is executed primarily through Hox genes or other miRNA targets.

## Conclusions

The survival of an organism depends on its ability to successfully and reproducibly execute a multitude of essential behaviors. This critically relies on Hox-dependent region-specific neuromuscular networks established along the AP axis of the body. Hox genes have been extensively investigated for their role in MN specification and motor circuit assembly in the hindbrain and the spinal cord of vertebrate CNS (Jessell, 2000; Philippidou and Dasen, 2013). The MNs in the hindbrain have a clustered organization, while in the spinal cord MNs are organized into longitudinal columns. At lower cervical (brachial) and lumbar levels of the spinal cord, MNs of the lateral motor column (LMC) project axons toward the forelimbs and hindlimbs (Landmesser, 2001). These columnar identities are regulated by the action of one or multiple Hox genes. Hox genes also diversify the MNs within LMC to generate approximately 50 MN pools targeting different limb muscles (Dasen et al., 2005). The cross-repressive interactions between different Hox genes set up a distinct transcriptional profile for each pool, which contributes to their clustering and peripheral muscle innervation (Dasen et al., 2008). Expectedly, individual Hox mutants in vertebrates affect the pool sizes, their position, and MN arborization. For example, in the case of HoxC6 mutants, brachial LMC size is reduced (Tiret et al., 1998; Vermot et al., 2005; Lacombe et al., 2013). Similarly, in the lumbar region where Hox10 is a major determinant of LMC identity, different mutant combinations for Hox10 result in defects in hindlimb innervation and compromise MN survival (Wahba et al., 2001; Lin and Carpenter, 2003; Shah et al., 2004; Wu et al., 2008). It has also been shown in the spinal cord MNs that acquisition of their basic MN identity and features is Hox independent (Jessell, 2000). These observations are reminiscent of the thoracic LinA/LinB lineage in *Drosophila*, which generate MNs innervating the adult leg muscles. In the case of LinB lineage, the Hox gene Pb and other mTFs play an instructional role in giving unique axonal and dendritic arborization to three MNs of the lineage, thereby regulating the morphological diversity of the MNs (Enriquez et al., 2015). Interestingly, Pb was not required for the survival of these MNs. This underlines the importance of Hox in determining the uniqueness of neuronal morphology. This genetic control of the morphological diversification of MNs was also shown to be critical in their functional capability for flawless walking at high speed (Baek and Mann, 2009; Baek et al., 2013; Enriquez et al., 2015). The role of Hox genes in determining the morphology of the vertebrate MNs has been reported. However, in our limited knowledge, no similar functional correlation between MN morphology and behavior has been established so far in the vertebrates. The observations made in thoracic LinA MNs are closer to what is reported in the vertebrates. In the case of *Antp* or the Hox triple (*Scr* ^*−*^, *Antp* ^*−*^, *Ubx* ^*−*^) mutants LinA NBs, MNs were reported to undergo apoptosis. When the cell death was blocked, the surviving neurons took their fate as thoracic MNs. These MNs innervate the right target muscles and exhibit subtle arborization defects (Baek et al., 2013). This was similar to what was reported in the case of vertebrate. However, unlike vertebrates, the majority of the *Drosophila* LinA MNs do not show expression of more than one Hox factor, or Hox gene cross-regulation playing a central role in determining MN identity (Baek et al., 2013). Only in the case of the T3 segment, LinA MNs express Antp in larval stages and Ubx in pupal and adult stages (Figures 2E–E') (Baek et al., 2013). The mutant analysis for these MNs suggested that Ubx expression represses Antp, and these two genes function redundantly in these cells of the T3 segment (Baek et al., 2013). None of the thoracic LinA MNs expressed Hox gene Pb (Baek et al., 2013). The apparent differences in the role of Hox genes in *Drosophila* compared to their elaborate role in specifying MN pool identity might be due to the complex limb musculature found in the vertebrates, which need a very refined control from MNs. It is reported that 11 Hox genes are required to diversify the MN pools, which innervate the muscles of anterior limbs alone (Dasen et al., 2005). On the other hand, *Drosophila* leg musculature is not as complex and therefore may not require such complex transcriptional code to generate a large diversity of MNs. However, all these conclusions in *Drosophila* and their comparisons with vertebrates are based on studies done in LinA and LinB lineages, which constitutes only two-third of the leg MNs. It is possible that detailed analysis of other leg innervating MNs in *Drosophila* may give some additional insights (Truman et al., 2004; Baek and Mann, 2009).

The other *Drosophila* studies discussed here (summarized in Table 1) highlight the importance of Hox genes in setting up a molecular code for the functional assembly of neuromuscular networks (Friedrich et al., 2016; Hessinger et al., 2017). These studies also established that the requirement of Hox genes in the cells is not transient and restricted to the formation of the networks, but is chronic and is required for the maintenance and functioning of the networks much after they are established (Friedrich et al., 2016). At the cellular level, Hox genes have been shown to play a role in the survival of the MNs (Baek et al., 2013), their muscle innervation, and in determining their axonal and dendritic morphology (discussed above) (Baek et al., 2013; Enriquez et al., 2015). Notably, studies (with Antp) also established that the level of Hox genes in the adult MNs could regulate their axonal targeting and innervation onto the muscles, with low expressing MNs targeting proximal leg muscles and vice versa (Baek et al., 2013). The studies with BX-C miRNA emphasized the importance of maintaining a fine control over Hox expression in the MNs to establish a functional neuromuscular network and its role in executing the behavior (Garaulet et al., 2014; Picao-Osorio et al., 2015; Issa et al., 2019). More specifically, the miRNA-mediated control of Ubx expression on the neural activity of the SR MNs was the first instance where fine control over the levels of Hox gene has been shown to impact both neurophysiology and behavior. How exactly is this effect executed in MNs, and whether the miRNA-mediated regulation of Hox levels impacts other adult behaviors remains to be addressed.

Many roles discussed here go beyond the conventional developmental roles played by Hox genes in AP axis determination. These studies establish that in addition to giving the neurons their positional identity and the capacity to form the region-specific neural circuitry, Hox genes have a functional requirement in adult stages in regulating, at the very least, the morphology and neural activity of the MNs and their functions. Therefore these functions, to some extent, explain the sustained and robust neuronal expression of these genes post differentiation (Hirth et al., 1998; Technau et al., 2006). In order to further understand the role of Hox genes in the assembly of neuromuscular networks along the AP axis as well as their function beyond, there is a need to identify their targets in MNs. For instance, both Hox and Hth were similarly required for thoracic MNs to survive (Baek et al., 2013), but phenotypes like axonal and dendritic morphology differed when either Hox or Hth were individually removed (Baek et al., 2013; Enriquez et al., 2015). This supported the idea that distinct genetic programs downstream of Hox and Hth control axonal and dendritic morphology independent of each other. Therefore, identifying Hox and Exd/Hth targets specifically in MNs will be useful to understand their role in neuromuscular circuit assembly and their morphological diversification. Hox target genes have been identified in past using various approaches (Leemans et al., 2001; Barmina et al., 2005; Mohit et al., 2006; Hersh et al., 2007; Hueber et al., 2007; Agrawal et al., 2011; Choo et al., 2011; Pavlopoulos and Akam, 2011; Slattery et al., 2011; Sorge et al., 2012; Beh et al., 2016; Prasad et al., 2016; Shlyueva et al., 2016). However, none of these approaches were geared towards identifying the targets within CNS or its specific cell types. Identifying Hox targets in MNs may have been technically difficult so far, but a finer refinement of targeted DamID (TaDa) (Southall et al., 2013) to an elegant nano DAM technique (https://www.biorxiv.org/content/10.1101/2021.06.07.447332v2) may provide a useful mean for identifying MN specific targets genes downstream to these factors. Lastly, the distinct morphological phenotypes observed in MNs in *Antp* and *hth* mutants also suggest that Antp may be using cofactors other than canonical Hox cofactors (like Exd and Hth) (Baek et al., 2013). This is not unusual as Hox genes have been shown to use cooperative co-factors other than Exd/Hth (Gebelein et al., 2004; Ghosh et al., 2019), as well as novel collaborative co-factors in both neural and non-neural cell types (Gebelein et al., 2004; Mann et al., 2009; Baëza et al., 2015; Khandelwal et al., 2017; Bischof et al., 2018; Ghosh et al., 2019; Bakshi et al., 2020; Carnesecchi et al., 2020). However, the question remains whether any of these non-canonical cofactors facilitate Hox genes to carry out their conventional and newly discovered roles in MNs.
